# Identification of the immunosuppressive effect of γδ T cells correlated to bone morphogenetic protein 2 in acute myeloid leukemia

**DOI:** 10.3389/fimmu.2022.1009709

**Published:** 2022-10-17

**Authors:** Shuang Liang, Tianhui Dong, Keli Yue, Haitao Gao, Ning Wu, Ruoyang Liu, Yan Chang, Le Hao, Lijuan Hu, Ting Zhao, Qian Jiang, Xiao-Jun Huang, Jiangying Liu

**Affiliations:** ^1^ Peking University People’s Hospital, Peking University Institute of Hematology, National Clinical Research Center for Hematologic Disease, Beijing Key laboratory of Hematopoietic Stem Cell Transplantation, Beijing, China; ^2^ Peking-Tsinghua Center for Life Sciences, Academy for Advanced Interdisciplinary Studies, Peking University, Beijing, China

**Keywords:** acute myeloid leukemia, immune suppression, γδ T cells, bone morphogenetic protein, immunotherapy

## Abstract

Description of immune landscapes in malignant microenvironment is critical to the improvement of therapeutic strategies for various tumors. Acute myeloid leukemia (AML) remains a severe life-threatening malignancy and often confronts treatment dilemma in clinic. Although γδ T cells exhibit independent and potent cytotoxicity against leukemic cells *in vitro* and in the mouse models, efficacy of γδ T cell-based immunotherapy on AML patients has seemed unsatisfying so far. How the anti-AML capacity of γδ T cells is suppressed *in vivo* remains elusive. Herein, we found an aberrant γδ T cells subset expressing CD25^+^CD127^low^Vδ2^+^ in the bone marrows of patients with newly diagnosed AML. The emergence of this subset was significantly associated with disease status and risk stratification as well as with the abnormally increased bone morphogenetic protein 2 (BMP2). Mechanistically, BMP2 could directly induce CD25^+^CD127^low^Vδ2^+^ γδ T cells (named as Reg-Vδ2) *in vitro*. The immunosuppressive features of Reg-Vδ2 cells were identified by combining immunophenotypical and functional data. Furthermore, inhibition of BMP2 pathway significantly blocked the emergence of Reg-Vδ2 cells and enhanced the anti-AML immunity in humanized mice. These findings not only provide a novel insight into the mechanisms of immunosuppression in the context of leukemia, but also suggest potential targets for the treatment of AML and other hematopoietic malignancies.

## Introduction

Despite the understanding about leukemia biology and the therapeutic strategies for leukemia have improved over the past decades, patients with acute myeloid leukemia (AML) generally experienced dismal outcome (1, 2). Treatment of patients with relapsed/refractory AML remains a big challenge in clinic (3). γδ T cells have drawn increasing attentions due to their powerful anti-tumor effects on both solid and hematologic malignancies (4, 5). A meta-analysis study reported that tumor-infiltrating γδ T cells were the most significant favorable immune cell population for prognostic association with 25 human cancers (6). Vδ2^+^ T cells, as a dominant subpopulation of peripheral γδ T cells, can be specifically activated by phosphoantigens and expand upon the treatment of aminobisphosphonates. Previous studies showed that the expanded Vδ2^+^ T cells exhibited direct and potent cytotoxicity against leukemia cells *in vitro* and in the mouse models, which were independent of MHC molecules and other antigen-presenting cells (7–10). However, efficacy of clinical investigations using Vδ2^+^ T cell-based approaches for the treatment of AML has seemed unsatisfying so far (11). How the anti-leukemia capacity of γδ T cells is restrained in the scenario of AML is unknown.

Recent studies have demonstrated that γδ T cells displayed pro-tumor capacities in some solid tumors (12, 13). Typically, interleukin 17 (IL-17)-producing γδ T cells exerted pro-tumor function through recruitment of myeloid-derived suppressor cells in human colorectal cancer and regulated neutrophil accumulation, phenotypes, and metastasis in breast cancer (14, 15). Another study showed that γδ T cells producing regulatory cytokines promoted pancreatic oncogenesis (16). Whether γδ T cells exhibit leukemia-promoting features and the related mechanisms remain unclear. A few studies observed some abnormal phenotypes of γδ T cells in peripheral blood of AML patients (17–19), but the phenotype-related functions and the picture in AML bone marrow niche have not been described. For better exploiting their potent anti-leukemia effect, it is necessary to dissect γδ T subsets with differential functions and the specific components that may regulate the functional transformation of γδ T cells in the microenvironment of AML.

Bone morphogenetic proteins (BMPs) belong to a superfamily of transforming growth factor-β (TGF-β) and function in cell migration, apoptosis, and differentiation of multiple tissues (20, 21). The dysregulation of BMP pathway was previously reported in acute promyelocytic leukemia and chronic myeloid leukemia (22–24). Recent investigations demonstrated the implication of BMP family members in regulating the survival and stemness of AML blasts (25, 26). With respect to the modulation on T cells, a recent study found that BMPs and BMPR1a signaling controlled Treg cells through impacting Foxp3 expression (27). However, such regulation has not been described in any disease conditions. The effect of BMPs on the phenotypes and functions of γδ T cells remains to be investigated. Notwithstanding TGF-β was reported to trigger the aberrant changes of γδ T cells *in vitro* (28, 29), this phenomenon has not been found in the context of AML.

In the current study, we sought to explore the abnormal phenotype and functional activity of γδ T cells that could attenuate their anti-AML effect and whether this change was correlated with some environmental factors such as TGF-β family members. Whether blocking BMP pathway restores or enhances the anti-AML capacity of γδ T cells was also investigated. Our findings may reveal a novel mechanism underlying the immunosuppression in AML and develop effective γδ T cells-based strategies for immunotherapy of AML patients.

## Methods

### Donors and patients

Bone marrow samples were obtained from 62 adult patients with newly diagnosed AML excluding promyelocytic leukemia and 51 healthy donors during routine diagnostic and transplantation procedures at Peking University Institute of Hematology from August 2020 to June 2021. The general clinical characteristics were summarized in **Supplementary Table 1**. The AML subtypes were classified according to World Health Organization (WHO) classification (30). Risk stratification (including favorable, intermediate and poor risk categories) for included patients was based on the cytogenetics and molecular abnormalities defined according to 2017 European LeukemiaNet (ELN) recommendations (31) and National Comprehensive Cancer Network (NCCN) Guidelines Version 3.2021 for Acute Myeloid Leukemia. The Ethics Committee of Peking University People’s Hospital approved the study protocol. All donors and patients signed consent forms.

### Immunophenotyping

Briefly, fresh bone marrow samples were stained with the following fluorochrome-labeled antibodies: PE-Cy7 anti-CD3, BV510 anti-TCRαβ, PE anti-Vδ2, APC-Cy7 anti-NKG2D, BV711 anti-PD-1, PE-Cy5 anti-CD25, and BV421 anti-CD127 (BioLegend, USA), and FITC anti-Vδ1 (Thermo Scientific, USA). Polychromatic flow cytometric analyses were performed on a BD LSRFortessa™ Cell Analyser and further analyzed using BD FACSDiva™ software.

### Elisa assays

The supernatants of bone marrows were freshly collected from healthy donors and newly diagnosed AML patients, and the equal volume of supernatants were detected using ELISA kits (4A Biotech, China) respectively for human TGF-β, BMP2, BMP4, and ActivinA, according to the manufacturer’s instructions. For the detections of cytokines from *in-vitro* expanded Vδ2 cells, equal amount of effector and regulatory Vδ2 cells were washed and plated into fresh media at the peak day of expansion, followed by culture for another 5 days. Then the supernatants were collected and detected using ELISA kits (4A Biotech, China) respectively for human IFN-γ, TGF-β, IL-1β, IL-4, IL-10, and IL-17, according to the manufacturer’s instructions.

### *In-vitro* stimulation and expansion of CD25^+^CD127^low^ Vδ2^+^ T cells

For short-term stimulation, peripheral blood mononuclearcells (PBMCs) from healthy donors were treated with BMP2 (50 ng/mL), with or without Pamidronate (Shenzhen Neptunus Pharmaceutical Co., China, 9 μg/mL) for 48 hours. For ex-vivo expansion of CD25^+^CD127^low^ Vδ2^+^ T cells, PBMCs from healthy donors (4×10^6^ cells/mL) were cultured in RPMI 1640 media supplemented with 10% FBS and recombinant human BMP2 (50 ng/mL), IL-2 (25 ng/mL), and IL-15 (50 ng/mL). Cytokines were purchased from Stemimmune LLC, USA. Pamidronate (9 μg/mL) was added only at the first day. Every 3 days, half volume of the supernatants was replaced with fresh media containing cytokines. After 10 days of culture, the proportion of Reg-Vδ2 cells was detected by flow cytometry. Then cells were stained with fluorochrome-labeled anti-CD3, anti-TCRVδ2, anti-CD25, and anti-CD127 antibodies and Reg-Vδ2 cells (CD3^+^Vδ2^+^CD25^+^CD127^low^) sorted by flow cytometry.

### *In-vitro* expansion of effector Vδ2 cells

To compare the expansion ability of intrinsic Vδ2 cells, mononuclear cells were isolated from bone marrow samples of healthy donors and AML patients at diagnosis. Isopentenyl pyrophosphate triammonium salt solution (IPP, Sigma) was added to a final concentration of 20 μg/mL at the day 0. Recombinant human interleukin-2 (Stemimmune LLC, USA) was added to a final concentration of 50 ng/mL every third day since day 3.

To get large amount of effector Vδ2 cells used in a series of functional experiments, PBMCs were isolated from healthy donors. The isolated mononuclear cells (2×10^6^ cells/mL) were cultured in RPMI 1640 media. Pamidronate was added to a final concentration of 9 μg/mL at the day 0 and day 3. Recombinant human interleukin-2 was added to a final concentration of 50 ng/mL every third day since day3. By 12–14 days of culture, the proportion of Vδ2^+^ T cells was detected by flow cytometry analysis. Then Vδ2^+^ T cells were purified by FITC-TCRVδ2 antibody (Biolegend) and anti-FITC MicroBeads (Miltenyi Biotech, Germany).

### Cytotoxicity assay

Pamidronate expanded effector Vδ2 (Eff-Vd2) and Reg-Vδ2 cells were purified (purity > 90%) as described above. Reg-Vδ2 cells were pre-labelled with calcein-AM (100 nM, BioLegend, USA) and co-cultured with Eff-Vδ2 for 48 hours, at the indicated ratios respectively. U937 cells were added afterwards and cultured for additional 4 hours, at 1:10 ratio versus Eff-Vδ2 cells. Then T cells were stained with the fluorochrome-labeled anti-CD3, anti-Vδ2, anti-CD25, anti-CD127, anti-CD38, anti-DNAM1, and anti-NKG2D. Detection for the expression of IFN-γ in Vδ2 cells was same as our previous report (32). U937 cells were detected by 7-AAD staining (BioLegend, USA).

### Mice

Severe immunodeficient NOD-Prkdc^scid^ Il2rg^tm1^/Vst (NPG) mice were purchased from Beijing Vitalstar Biotechnology (China). To investigate the direct effect of Reg-Vδ2 cells on the anti-AML function of effector Vδ2 cells, 6-7 week-old female NPG mice were intravenously injected with luciferase and GFP co-expressed U937 cells (6×10^4^/mouse) on day 0. Eff-Vδ2 (12×10^6^/mouse) and Reg-Vδ2 cells (2.4×10^6^/mouse) were intravenously injected on days 1 and 3.

To establish a humanized mouse model, 5-6 week-old female NPG mice were irradiated at a dose of 1 Gy. Then purified human CD34^+^ cells from healthy donors were injected into the mice (5 x 10^5^ cells/mouse) *via* the tail vein. At 6-7 weeks after injection, PB samples were collected from mice and detected by flow cytometry using BV711 anti-mouse CD45 and PerCP anti-human CD45 antibodies (BioLegend). The successful implantation of human white blood cells was confirmed as the percentage of human CD45-positive cells in mouse peripheral mononuclear cells was > 20%. In view of the existence of human T cells in mice, a little higher dose (1 x 10^5^) of U937 cells co-expressing luciferase and GFP was intravenously injected into the humanized mice. Intraosseous injections of 20 μL of BMP2 (1 ng/μL/leg) and/or k02288 (25 ng/μL/leg) were performed at 1, 7, 14, and 21 days after U937 injection.

Establishment and implantation of luciferase-expressing U937 cells allowed us to monitor the growth of human AML cells *in vivo* by noninvasive whole-body imaging. Our pilot experiments showed that the extensive distribution of luciferase-expressing cells could be detected around 15 to 20 days after injection. Mice with hind-leg paralysis were counted as death and were sacrificed. Bone marrow samples were collected immediately after scarification and the mononuclear cells were detected by flow cytometry. The co-expression of GFP on U937 cells was utilized to detect the frequency of human AML cells in the bone marrows of mice. The frequencies of implanted humanVδ2 cells and the emergence of Reg-Vδ2 cells in mice bone marrows were detected using fluorochrome-labeled specific antibodies as described above. The survival of mice was monitored until the ends of different experiments.

### Statistical analysis

Statistical differences in proportions of γδ T cell subsets between donors and AML patients were analyzed using the non-parametric (Mann-Whitney) t test. Comparisons between the indicated two groups in *in-vitro* experiments were analyzed using Student’s t-test. Statistical differences among more than two groups were analyzed using Kruskal-Wallis H-test and one-way analysis of variance (ANOVA). Data are presented in mean+/-SEM. The survival rates of mice were analyzed using Kaplan-Meier analysis. Statistical significance was defined as *P* ≤ 0.05, based on a two-tailed test. All calculations were performed using SPSS 22.0 statistical software (SPSS Inc, USA). Figure creations were performed using GraphPad Prism 8.0.2 (GraphPad Software, Inc., La Jolla, USA). Experimental graphics were generated with BioRender (https://biorender.com).

## Results

### CD25^+^CD127^low^Vδ2^+^ T cells were significantly increased in the bone marrows of AML patients

**Figure 1:**
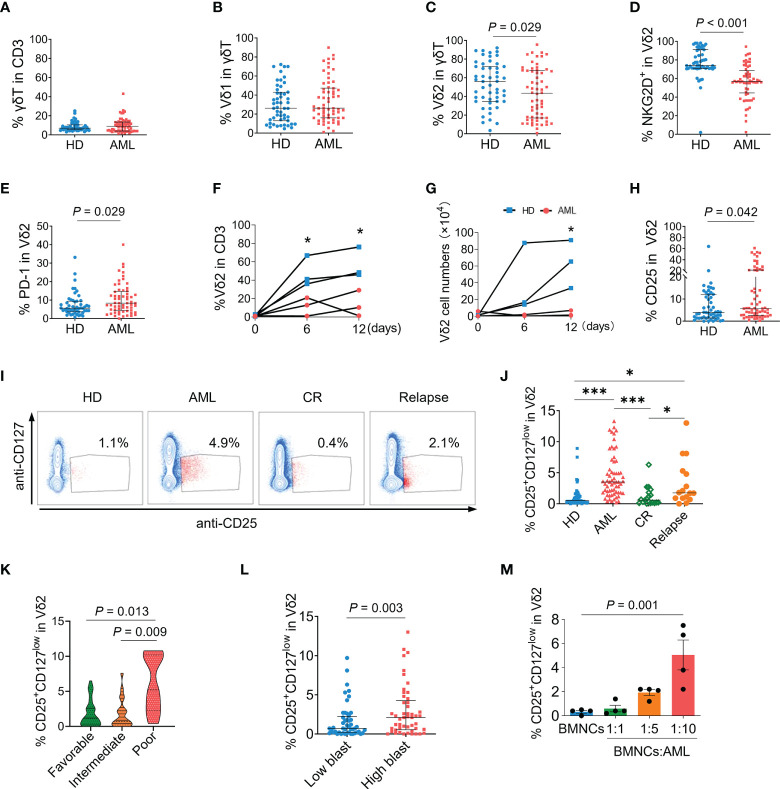
Immunophenotypes of γδ T cells and association of CD25^+^CD127^low^Vδ2^+^ T cells with AML. Flow cytometry analyses for γδ T cells in the bone marrow samples from newly-diagnosed AML patients (n = 62), compared with healthy donors (HD, n = 51). **(A)** The proportions of total γδ T cells in CD3^+^ T cells. **(B, C)** The proportions of Vδ1 and Vδ2 subsets in γδ T cells. Expressions of NKG2D **(D)**, and PD-1 **(E)** on Vδ2 T cells. Bone marrow mononuclear cells (BMNCs) isolated from healthy donors and AML patients were stimulated with IPP for indicated time course. The proportions of Vδ2 cells in CD3^+^ T cells **(F)** were detected by flow cytometry and the absolute numbers of Vδ2 cells were counted **(G)**. Expressions of CD25 **(H)** on Vδ2 T cells. Representative dot-plot images by flow cytometry **(I)** and the statistical analyses **(J)** of the frequency of CD25^+^CD127^low^Vδ2^+^ T cells in bone marrow samples from healthy donors, and AML patients at diagnosis, complete remission (CR) after chemotherapy (n = 19), and relapse (n = 16). *P < 0.05, **P < 0.01, ***P < 0.001. **(K)** The frequencies of CD25^+^CD127^low^Vδ2^+^ T cells in bone marrows of newly-diagnosed AML patients with favorable (n = 34), intermediate (n = 19), and poor (n = 9) risk status. **(L)** Comparison of the frequency of CD25^+^CD127^low^Vδ2^+^ T cells in patients with ≤median (low) and > median (high) load of AML blast. **(M)** The proportions of CD25^+^CD127^low^Vδ2^+^ T cells in Vδ2 T cells after co-culture of primary AML cells isolated from patients with BMNCs from healthy donors at indicated ratios for 48 hours. n = 4. Statistical differences in **(A–H)** and **(L)** were analyzed using the non-parametric (Mann-Whitney) t test, **(I)** and **(K)** using the Kruskal-Wallis H-test, and **(M)** using the one-way analysis of variance (ANOVA). *P* values are indicated on the graphs.

Since bone marrow is the primary tumor foci of AML, we initially analyzed the immunophenotypes of γδ T cells in the bone marrows of patients with newly diagnosed AML. The gating strategies of flow cytometry analyses for γδ T subsets and functional markers are shown in **Supplementary Figure S1A**. The proportions of total γδ T cells in CD3^+^ T cells and Vδ1^+^ fraction in γδ T cells were not significantly different between AML patients and healthy donors (*P* = 0.839 and *P* = 0.323, **Figures 1A, B**). In contrast, the percentage of Vδ2^+^ T cells was markedly decreased in AML patients (*P* = 0.029, **Figure 1C**). Meanwhile, a key activating receptor of γδ T cells, NKG2D, was dramatically decreased (*P* < 0.001, **Figure 1D**). Whereas the expression of regulatory receptor PD-1 (*P* = 0.029, **Figure 1E**) was increased on Vδ2^+^ T cells in AML patients. In response to the specific stimulation of IPP, the expansion capacity of primary Vδ2^+^ T cells in bone marrow mononuclear cells from AML patients was profoundly impaired compared to that from healthy donors (*P* < 0.05, **Figures 1F, G**). These results demonstrated the functional abnormalities of Vδ2^+^ T cells in the bone marrows of AML patients. Meanwhile, more Vδ2^+^ T cells from AML patients expressed CD25 in contrast to those from healthy donors (*P* = 0.042, **Figure 1H**), indicating a regulatory fraction of γδ T cells may exist in the context of AML.

Since the expression of Foxp3 on γδ T cells was almost undetectable in both healthy and AML groups in the present study (data not shown), combining detection of CD25 and CD127 expression was utilized to identify the regulatory phenotype of Vδ2^+^ T cells. As shown in **Figures 1I, J**, a subset expressing CD25 and low CD127 (CD25^+^CD127^low^) was emerged in Vδ2^+^ T cells of AML patients compared with healthy donors (4.9% versus 1.1%, *P* < 0.001). The proportions of CD25^+^CD127^low^ fraction in Vδ1 and non-Vδ1Vδ2 cells were not significantly increased when comparing AML patients versus healthy donors (**Supplementary Figures S2A, B**).

In contrast to AML patients at diagnosis, the proportion of CD25^+^CD127^low^Vδ2^+^ T cells was dramatically decreased in AML patients at completed remission (0.4% vs 4.9%, *P* < 0.001), which was also significantly lower than that in AML group at relapse (0.4% vs 2.1%, *P* < 0.05, **Figures 1I, J**). Notably, the frequency of CD25^+^CD127^low^Vδ2^+^ T cells was dramatically higher in patients with poor-risk AML than those with favorable- and intermediate-risk AML (*P* = 0.013 and *P* = 0.009, **Figure 1K**). Consistently, the frequency of CD25^+^CD127^low^Vδ2^+^ T cells was significantly increased in AML patients with higher blast load (*P* = 0.003, **Figure 1L**). To confirm CD25^+^CD127^low^Vδ2^+^ T cells were specifically induced in the environment of AML, bone marrow mononuclear cells (BMNCs) isolated from healthy donors were co-cultured with purified primary AML cells from patients at indicated ratios. Compared to the non-AML group, the proportion of CD25^+^CD127^low^Vδ2^+^ T cells was continuously elevated following the presence of primary AML cells at increasing ratios (**Figure 1M**). While lower general proportion was observed in PB than that in BM, the frequency of this aberrant Vδ2 subset was significantly higher in PB of AML patients compared with that of healthy donors (**Supplementary Figure S2C**). These results demonstrated that the emergence of CD25^+^CD127^low^Vδ2^+^ T cells was a specific immunophenotype in AML and correlated with disease status and prognosis.

### The concentration of BMP2 was remarkably elevated and positively correlated with the frequency of CD25^+^CD127^low^Vδ2^+^ T cells in the bone marrows of AML patients

**Figure 2:**
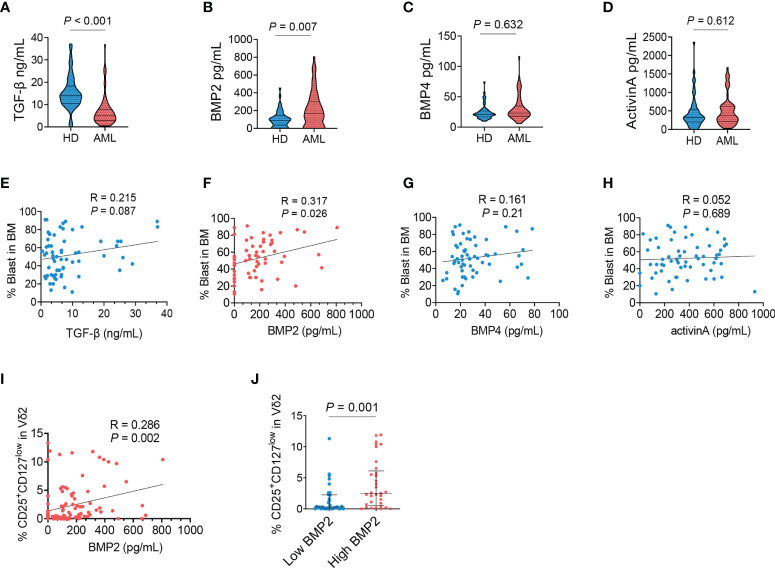
Dysregulated BMP2 in AML patients and its correlation with CD25^+^CD127^low^Vδ2^+^ T cells. The supernatants of bone marrows collected from healthy donors and AML patients were detected using ELISA kit for TGF-β **(A)**, BMP2 **(B)**, BMP4 **(C)** and ActivinA **(D)**. Correlation analyses for the levels of TGF-β **(E)**, BMP2 **(F)**, BMP4 **(G)**, and ActivinA **(H)** with the load of AML blasts (n = 62). **(I)** Correlation analyses for the proportions of CD25^+^CD127^low^Vδ2^+^ T cells with the levels of BMP2 in AML patients and healthy donors. *P* values are shown on the graphs respectively. **(J)** Comparison of the frequency of CD25^+^CD127^low^Vδ2^+^ T cells in AML patients with ≤median (low) and > median (high) concentrations of BMP2. *P* values are shown on the graphs respectively.

We questioned what factors mediated the abnormal phenotype of γδ T cells in AML. TGF-β was first considered due to its immunoregulatory effect especially on the development and differentiation of T cells. However, the levels of TGF-β were dramatically decreased in the bone marrows of AML patients (*P* < 0.001, **Figure 2A**). Then the detection was extended to other members of TGF-β superfamily. We found that the concentration of BMP2 was markedly elevated in AML patients compared with healthy donors (*P* = 0.007, **Figure 2B**). Whereas the levels of BMP4 (*P* = 0.632, **Figure 2C**) and ActivinA (*P* = 0.612, **Figure 2D**) were comparable between the two groups. A positive association was found between the frequency of AML blasts and the level of BMP2 (*P* = 0.026, **Figure 2F**), but not the levels of TGF-β (*P* = 0.087, **Figure 2E**), BMP4 (*P* = 0.210, **Figure 2G**), and ActivinA (*P* = 0.689, **Figure 2H**). Correlation analysis further demonstrated that the frequency of CD25^+^CD127^low^Vδ2^+^ T cells was positively associated with the levels of BMP2 in bone marrows (*P* = 0.002, **Figure 2I**), which was confirmed by that more CD25^+^CD127^low^Vδ2^+^ T cells were found in AML patients with higher concentration of BMP2 (*P* = 0.001, **Figure 2J**). Collectively, these results demonstrated that the emergence of CD25^+^CD127^low^Vδ2^+^ T cells was significantly correlated with the abnormally elevated BMP2 levels in AML patients.

### BMP2-induced CD25^+^CD127^low^Vδ2^+^ T cells exhibited distinct immunophenotypes from effector Vδ2^+^ T cells

**Figure 3:**
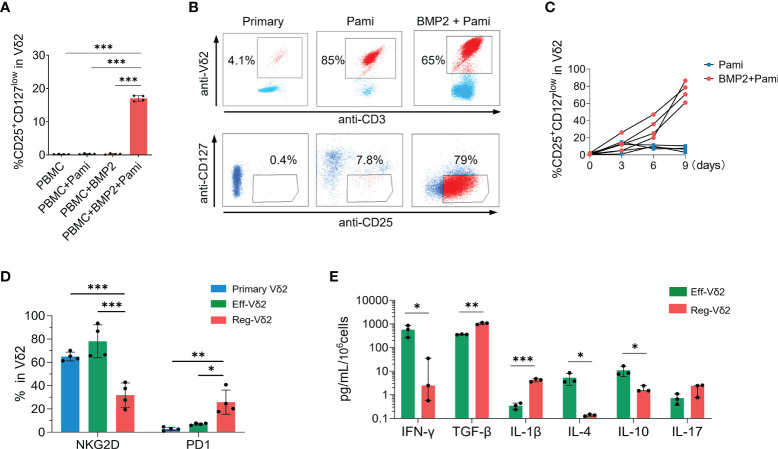
Immunophenotypes of CD25^+^CD127^low^Vδ2^+^ T cells. **(A)** The proportions of CD25^+^CD127^low^Vδ2^+^ T cells in Vδ2 T cells were detected in PBMCs isolated from healthy donors after short-time stimulation with pamidronate with or without BMP2. **(B)** PBMCs isolated from healthy donors were used to expand Reg-Vδ2 under conditions as described in Methods. Flow cytometry analyses showing the representative dot-plot images and proportions of Vδ2 in CD3^+^ T cells and CD25^+^CD127^low^ fraction in Vδ2 cells at primary state and after stimulation of pamidronate with or without BMP2. **(C)** The statistical analyses of percentage of CD25^+^CD127^low^Vδ2^+^ T cells (Reg-Vδ2) in pamidronate and BMP2 plus pamidronate groups. **(D)** Expressions of NKG2D and PD1 in primary, Eff-Vδ2 (Eff-Vδ2 cells were expanded as described in Methods), and Reg-Vδ2 cells were detected by flow cytometry. n = 4, *P < 0.05, ***P < 0.001. **(E)** Secretions of IFN-γ, TGF-β, IL-1β, IL-4, IL-10, and IL-17 from Eff-Vδ2 and Reg-Vδ2 cells were detected by ELISA assays. n = 3, *P < 0.05, **P < 0.01, ***P < 0.001.

In line with the correlation described above, *in-vitro* short-time stimulation with BMP2 plus pamidronate directly induced the CD25^+^CD127^low^ fraction of Vδ2^+^ T cells isolated from healthy donors whereas pamidronate or BMP2 alone did not (*P* values < 0.001, **Figure 3A**). To further demonstrate the immunophenotypes and functions of CD25^+^CD127^low^Vδ2^+^ T cells, we expanded this special subset from healthy PBMCs in the presence of BMP2 and pamidronate complemented with necessary cytokines. After culture for 9 days *in vitro*, the proportion of CD25^+^CD127^low^ fraction in Vδ2 cells was dramatically increased compared with those at primary status and with treatment of pamidronate alone (**Figure 3B**), which was confirmed in PBMCs isolated from 4 independent healthy donors (**Figure 3C**). Such BMP2-expanded CD25^+^CD127^low^Vδ2^+^ T cells are abbreviated to regulatory Vδ2 cells (Reg-Vδ2) hereafter. The effector Vδ2 cells were expanded from PBMCs of healthy donors by stimulation with pamidronate (referred to Eff-Vδ2). Significantly lower NKG2D (*P* < 0.001) and higher PD-1 (*P* < 0.05) expressions were found in Reg-Vδ2 cells, compared with primary Vδ2 and Eff-Vδ2 cells (**Figure 3D**). These findings were consistent with the phenotypes of Vδ2^+^ T cells observed in AML patients. Then we determined the cytokines production of Reg-Vδ2 cells. Compared with Eff-Vδ2 cells, the secretion of IFN-γ was markedly decreased whereas more TGF-β and IL-1β were produced in Reg-Vδ2 cells. The levels of IL-4 and IL-10 were significantly decreased, but IL-17 levels were comparable between two groups (**Figure 3E**). These results indicated that Reg-Vδ2 cells produced some regulatory rather than inflammatory cytokines.

### Reg-Vδ2 cells suppressed the cytotoxicity of effector Vδ2 cells on both AML cell line and primary AML cells *in vitro*

**Figure 4:**
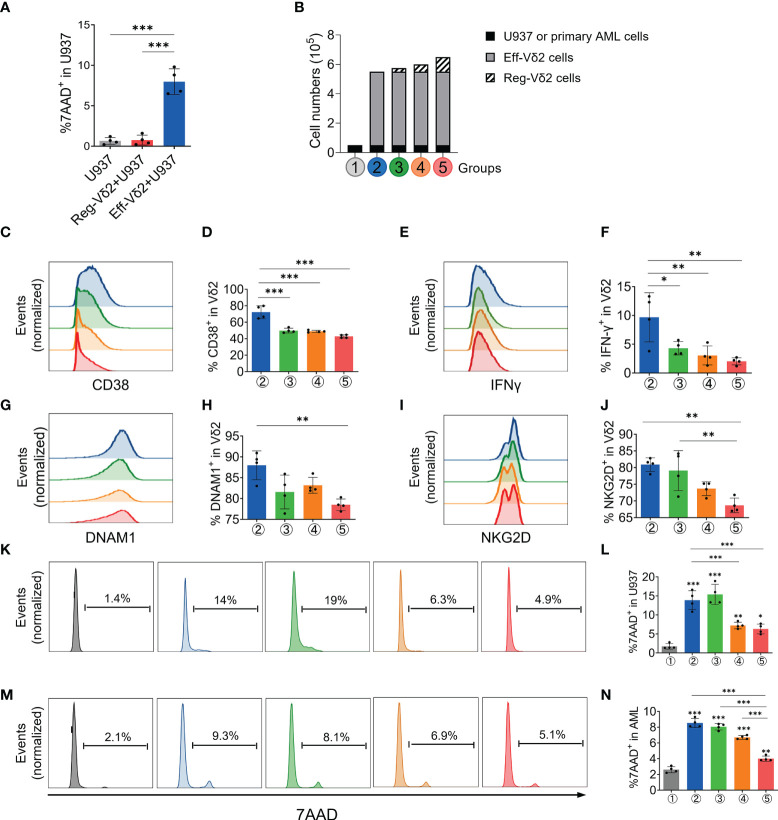
Impact of Reg-Vδ2 cells on the cytotoxicity of effector Vδ2 cells against AML *in vitro*. **(A)** Comparison of 7-AAD positive fractions in U937 cells after co-culture with Reg-Vδ2 and Eff-Vδ2 cells respectively. n = 4, ***P < 0.001. **(B)** Experimental groups showing that human AML cells (U937 cell line or primary leukemia cells isolated from patients with AML-M2, Group ①) were co-cultured with Eff-Vδ2 cells (Group ②), and with additional Reg-Vδ2 cells at ratios of 20:1 (Group ③), 10:1 (Group ④), and 5:1 (Group ⑤) respectively. Representative flow cytometry histograms and the statistical analyses for expressions of CD38 **(C, D)**, IFN-γ **(E, F)**, DNAM1 **(G, H)**, and NKG2D **(I, J)** in Vδ2 cells among the indicated groups. **(K, L)** Representative histograms of flow cytometry and the statistical analyses for 7-AAD positive fractions in U937 cells among the indicated groups. **(M, N)** Representative histograms of flow cytometry and the statistical analyses for 7-AAD positive fractions in primary AML cells among the indicated groups. Statistical differences were analyzed using the one-way analysis of variance (ANOVA). n = 4, *P < 0.05, **P < 0.01, ***P < 0.001.

Then we investigated whether Reg-Vδ2 cells functioned in the elimination of AML cells. In contrast to that Eff-Vδ2 cells induced a significant cell death of a human AML cell line, U937 cells (*P* < 0.001), the proportions of 7-AAD-positive U937 cells after treatment with Reg-Vδ2 cells were similar to the control (*P* > 0.1, **Figure 4A**). These results demonstrated that Reg-Vδ2 cells could not kill AML cells. To further investigate the regulatory effect of Reg-Vδ2 cells, U937 cells or primary leukemia cells isolated from AML patients (Group ①) were co-cultured with Eff-Vδ2 cells (Group ②). Calcein-AM pre-labelled Reg-Vδ2 cells were added at different ratios (Groups ③-⑤, **Figure 4B**). Flow cytometry analyses showed a significant downregulation of an activation marker, CD38, on Eff-Vδ2 cells after co-culture with Reg-Vδ2 cells (**Figures 4C, D**). The expressions of IFN-γ (**Figures 4E, F**), DNAM1 (**Figures 4G, H**), and NKG2D (**Figures 4I, J**) on Eff-Vδ2 cells were also decreased in the presence of Reg-Vδ2 cells. Consistently, the increased 7-AAD-positive U937 cells after co-culture with Eff-Vδ2 cells (from 1.4% to 13.6%, *P* < 0.001) were markedly declined (from 13.6% to 4.9%) by adding Reg-Vδ2 cells at increasing ratios (one way ANOVA analysis *P* < 0.001, **Figures 4K, L**). Similarly, the cytotoxic effect of Eff-Vδ2 cells on primary AML cells of patients was significantly impaired by Reg-Vδ2 cells (one way ANOVA analysis *P* < 0.001, **Figures 4M, N**). These results demonstrated that Reg-Vδ2 cells attenuated the anti-AML activity of effector Vδ2 cells *in vitro*.

### The immunosuppressive effect of Reg-Vδ2 cells was validated in mice

**Figure 5:**
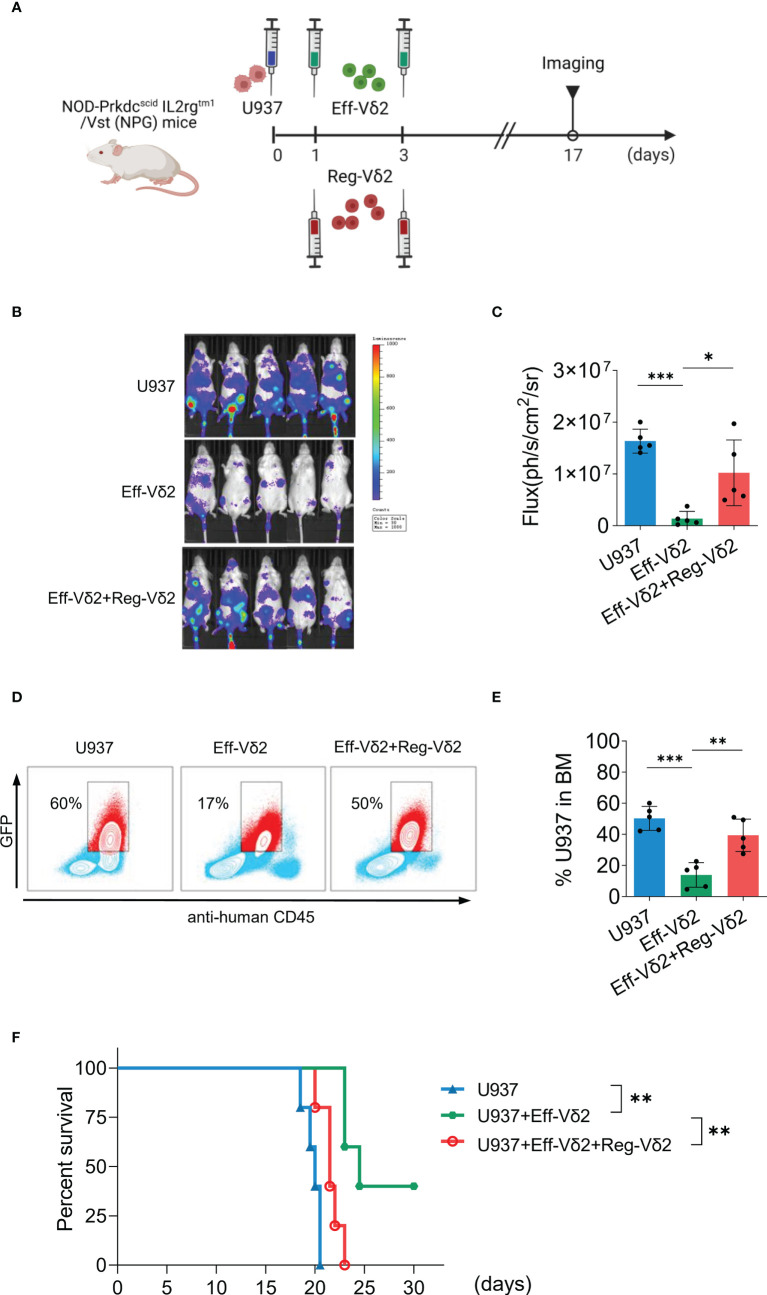
Impact of Reg-Vδ2 cells on the anti-AML activity of effector Vδ2 cells in mice. **(A)** Protocol for evaluating the direct influence of Reg-Vδ2 cells on the anti-AML activity of effector Vδ2 cells (Eff-Vδ2) in NPG mice. U937 cells co-expressing luciferase and GFP, and Eff-Vδ2 cells with or without Reg-Vδ2 cells were injected at indicated time points. Noninvasive whole-body imaging for the growth of U937 cells **(B)** and quantification of bioluminescence signal **(C)** in different groups of mice on day 17. **(D, E)** Representative dot-plot images of flow cytometry and the statistical analyses showing the frequencies of U937 cells in the bone marrows of mice with different treatments. **(F)** Survival of mice with different treatments until 30 days. n = 5, *P < 0.05, **P < 0.01, ***P < 0.001.

To validate Reg-Vδ2 cells exert immunosuppressive function *in vivo*, severe immunodeficient NPG mice were injected respectively with U937 cells co-expressing luciferase and green fluorescent protein (GFP)+PBS; U937+Eff-Vδ2 cells; and U937+Eff-Vδ2+Reg-Vδ2 cells at indicated time points (**Figure 5A**). Noninvasive *in-vivo* fluorescence imaging displayed that the growth of U937 cells was inhibited in mice treated with Eff-Vδ2 cells, compared to group only with U937 cells or combining treatment with Eff-Vδ2 and Reg-Vδ2 cells (**Figures 5B, C**). The gating strategies of flow cytometry analyses for GFP-positive U937 cells are shown in **Supplementary Figure S1B**. Injection of Eff-Vδ2 cells remarkably decreased the proportion of U937 cells (from 60% to 17%), whereas the frequency of U937 cells was maintained at 50% in the presence of Reg-Vδ2 cells (**Figures 5D, E**). Consequently, treatment with Eff-Vδ2 cells significantly prolonged the survival of mice implanted with human AML cells (*P* = 0.004, **Figure 5F**). Combining treatment with Reg-Vδ2 cells significantly impaired the protective effect of Eff-Vδ2 cells in AML mice (*P* = 0.003, **Figure 5F**). These results validated the immunosuppressive role of Reg-Vδ2 cells in restraining anti-AML activity of effector Vδ2 cells.

### Inhibition of BMP2 pathway facilitated the elimination of AML cells in humanized mice

**Figure 6:**
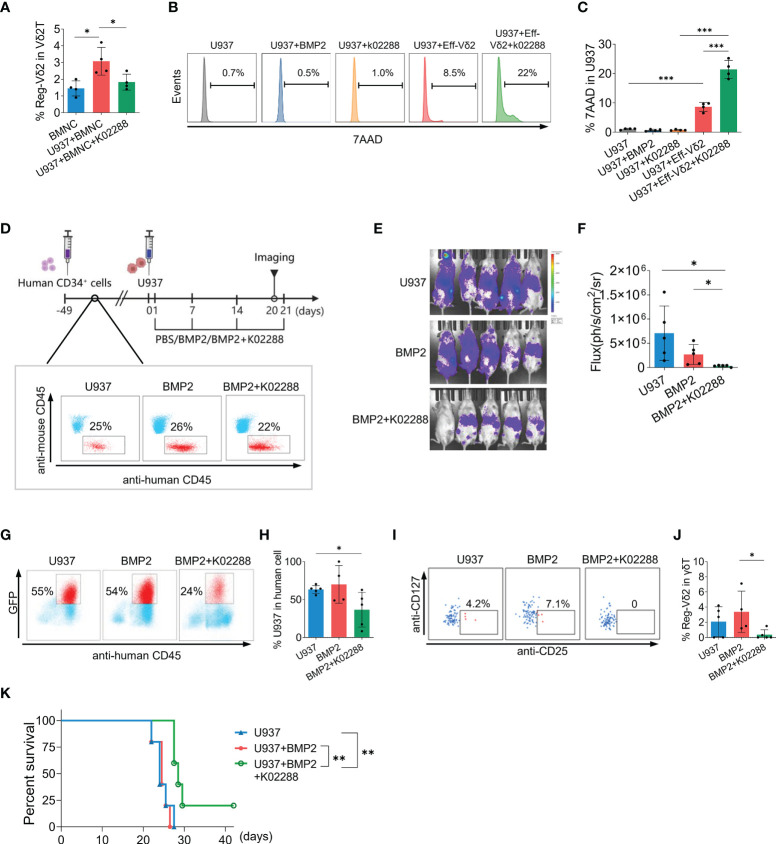
Effect of BMP-pathway inhibitor on the emergence of Reg-Vδ2 cells and the elimination of AML cells in humanized mice. **(A)** Bone marrow mononuclear cells (BMNCs) isolated from healthy donors were co-cultured with U937 cells, with or without an inhibitor of BMP receptor (k02288, 5 μmol/L). The frequencies of Reg-Vδ2 cells were detected by flow cytometry. n = 4, *P < 0.05. Representative histograms of flow cytometry **(B)** and the statistical analyses **(C)** for 7-AAD positive fractions in U937 cells among the indicated groups. n = 4, ***P < 0.001. **(D)** Protocol for evaluating the impact of k02288 on the anti-AML activity in humanized mice. NPG mice were initially injected with human CD34^+^ cells. Following implantation, U937 cells co-expressing luciferase and GFP, and BMP2 with or without k02288 were injected at indicated time points. Noninvasive whole-body imaging for the growth of U937 cells **(E)** and quantification of bioluminescence signal **(F)** among different groups on day 20. Representative dot-plot images of flow cytometry and the statistical analyses showing the frequencies of U937 cells **(G, H)** and Reg-Vδ2 cells **(I, J)** in the bone marrows of mice with different treatments. **(K)** Survival of mice with different treatments until 42 days. **(D–K)** n = 5, *P < 0.05, **P < 0.01.

Given the immunosuppressive roles of Reg-Vδ2 cells and BMP2 identified above, we further explored if blocking BMP2 pathway could restrain the emergence of Reg-Vδ2 cells and facilitate the anti-AML function of effector Vδ2 cells. The effects of a BMP pathway inhibitor, k02288, were initially investigated *in vitro*. As shown in **Figure 6A**, co-culture of U937 cells increased the frequency of Reg-Vδ2 cells in BMNCs from healthy donors, which was significantly decreased after treatment with k02288. Meanwhile, k02288 remarkably enhanced the cytotoxicity of Eff-Vδ2 cells against U937 cells (*P* < 0.001), whereas neither BMP2 nor k02288 itself caused the death of U937 cells (**Figures 6B, C**).

We next explored whether BMP2 pathway inhibitor relieved the immunosuppression on anti-AML activity *in vivo*. Following implantation with human CD34^+^ cells, NPG mice were randomly grouped and injected respectively with luciferase/GFP-expressing U937 cells+PBS; U937+BMP2; and U937+BMP2+k02288 at indicated time points (**Figure 6D**). Successful differentiation of human white blood cells was confirmed by flow cytometry analyses (**Figure 6D**). Whole-body imaging analysis showed that the injected AML cells extensively distributed in humanized mice with U937 or U937+BMP2. Whereas combining treatment with k02288 obviously restrained the growth of U937 cells (**Figures 6E, F**). Flow cytometry analyses for mouse BMNCs showed that the proportion of U937 cells was decreased after administration of k02288, compared with other groups (**Figures 6G, H**). While human Vδ2 cells were observed in the bone marrows of different groups by flow cytometry analyses, Reg-Vδ2 cells were detectable in U937 and U937+BMP2 groups, but not in U937+BMP2+k02288 groups (**Figures 6I, J**). As hypothesized, administration of k02288 significantly prolonged the survival of mice suffering AML, compared with groups of U937+BMP2 (*P* = 0.002) and U937 alone (*P* = 0.009, **Figure 6K**). The cumulative survival of mice with U937 was similar to U937+BMP2 group (*P* = 0.752, **Figure 6K**). Therefore, these results suggest that blockage of BMP2 pathway at least partially restores the intrinsic anti-AML immunity in humanized mouse models.

## Discussion

**Figure 7:**
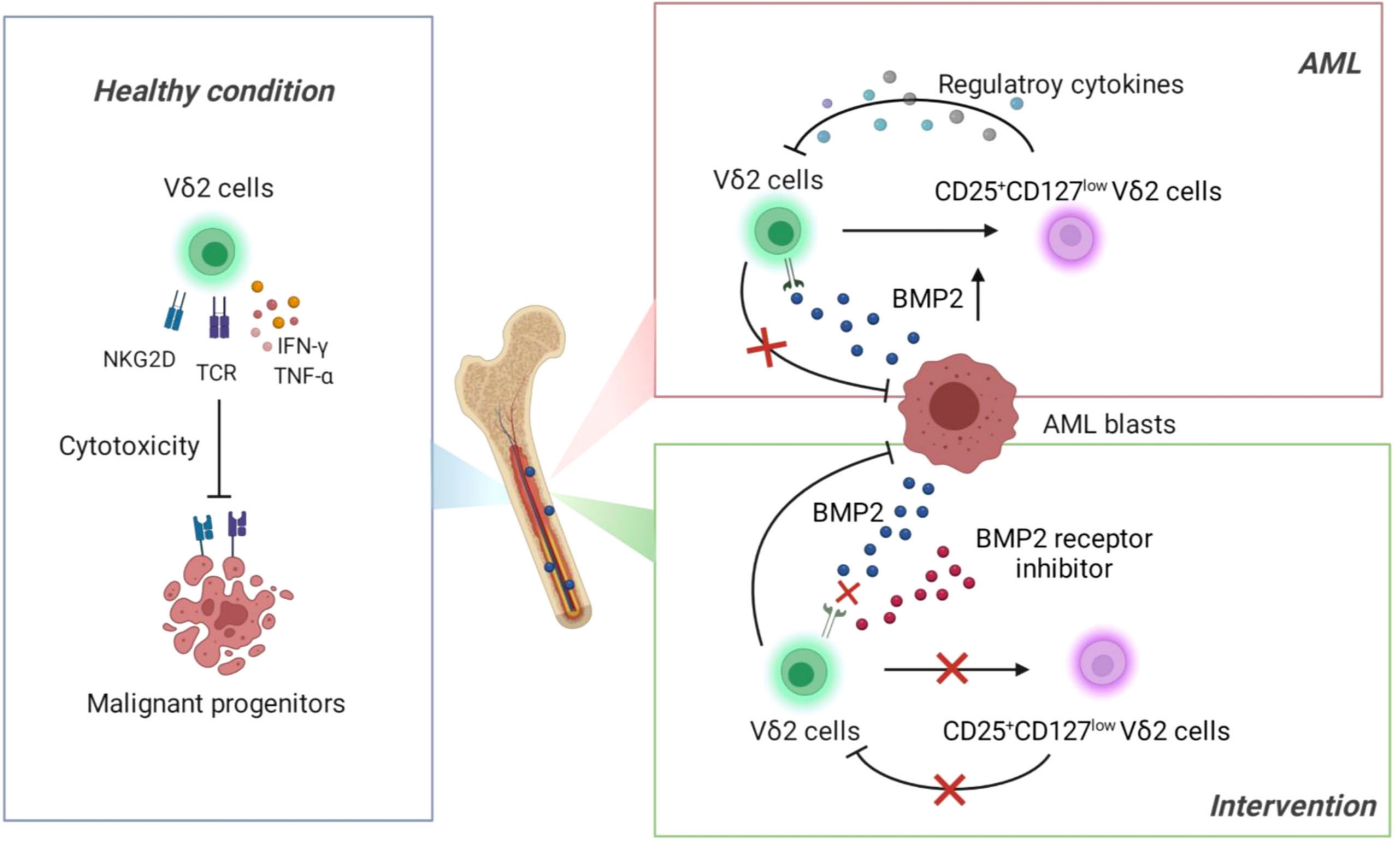
Schematic of main conclusions from this study.

Deficiency of T cells is a dominant immune landscape that closely links to the development of various malignancies (33, 34). Under healthy condition, γδ T cells exert first-line immune surveillance in the elimination of malignant cells through γδ T-cell receptor and other functional receptors (such as NKG2D) and cytokines (**Figure 7**, Left). This potent anti-tumor capacity without MHC restriction has made γδ T cells attractive in the establishment of novel immunotherapeutic strategies against solid and hematopoietic malignancies. However, recent studies identified a transformation of γδ T cells from warrior to foe during the development of some solid tumors (35, 36). So far it is elusive whether γδ T cells play a dual role in the context of leukemia. In the current study, we found that functional impairment of Vδ2 cells was accompanied with the emergence of an aberrant subset expressing CD25^+^CD127^low^Vδ2^+^ in the bone marrows of AML patients, which was significantly correlated with the abnormally increased BMP2 levels in this context. Subsequent functional experiments demonstrated that BMP2 could direct induce CD25^+^CD127^low^Vδ2^+^ T cells (named as Reg-Vδ2). The anti-AML activity of effector Vδ2 cells was remarkably suppressed by Reg-Vδ2 cells (**Figure 7**, Upper right). Inhibition of BMP pathway significantly attenuated the emergence of Reg-Vδ2 cells and enhanced the anti-AML capacity of effector Vδ2 cells (**Figure 7**, Lower right). Thus, our study uncovers the immunosuppressive feature of BMP2-induced γδ T subpopulation in the environment of AML, suggesting a novel mechanism accounted for the functional deficiency of intrinsic γδ T cells against AML.

Previous studies have reported the decreased proportion and abnormal phenotypes of γδ T cells in peripheral blood of AML patients (17). Given that bone marrow is the primary tumor foci of AML, exploration of the changes in immunophenotype of γδ T cells in bone marrow samples likely provides more significant immune characteristics previously uncovered in AML. In our present study, CD25^+^CD127^low^ served to define BMP2-induced Vδ2 cells that exhibited immunoregulatory features, which mirrored the definition and function of CD25^+^CD127^low^CD4^+^ T cells (regulatory T cells) in the literature (37, 38). We also determined the expression of Foxp3 in Reg-Vδ2 cells, unfortunately, our flow cytometry analyses using different brands of anti-Foxp3 antibodies all showed the extremely low expression of Foxp3 in γδ T cells of both healthy and AML cohorts (data not shown). Since TGF-β1 concentration was dramatically decreased in AML patients as shown in the current study (**Figure 2A**), our observation is consistent with the finding showing that FoxP3 expression in Vδ2 T cells could be induced by TGF-β1 (28). In addition, previous studies reported that TGF-β could enhance the cytotoxic activity of Vδ2 T cells through stimulating the productions of CD103 and IL-9 (39, 40). These findings imply that Reg-Vδ2 cells may not have the classic immunophenotype of CD4^+^ Tregs and their emergence is specific to the dysregulation of BMP2 in AML. Identification of other dominant markers for Reg-Vδ2 cells awaits future investigations.

It has been recognized that tumors are able to modify the functions of infiltrating immune cells and thereby create a favorable microenvironment for tumors (41, 42). In contrast to that TGF-β1 level in AML patients was much lower than that of healthy donors, we found that the concentration of BMP2 was abnormally upregulated, which was positively correlated with the frequency of leukemia blasts and the emergence of Reg-Vδ2 cells in the bone marrows of AML. Distinct from the findings in other studies that reported BMP2 functioned in the maintenance of leukemic stem cells (43, 44), our results unveiled that BMP2 regulated the phenotypic and functional changes of effector γδ T cells, leading to a suppressive effect on anti-AML immunity. The concentration of another BMP family member, BMP4, was reported to be elevated in the bone marrows of AML patients (n = 16) compared to healthy individuals (n = 20). Since we did not observe a significant increase in BMP4 levels from AML patients (n = 62, versus 51 healthy donors), whether BMP4 also triggers the phenotypic and functional transformation of γδ T cells was not the focus in our current study. Nevertheless, given that BMP family members share multiple receptors and pathways, it is unsurprising that BMP4 may converge with the immunomodulatory role of BMP2 in educating γδ T cells.

Multiple clinical trials utilized the expanded effector Vδ2 cells to treat patients with renal cell carcinoma, prostate cancer, cholangiocarcinoma, non-Hodgkin lymphoma, or multiple myeloma, and reported promising results (45–47). However, the efficacy of γδ T cells-based immunotherapy in the treatment of AML seems disappointing, compared to the striking results shown *in vitro* and in the mouse models (48). Clarification of this discrepancy relies on in-depth knowledge about the precise features of γδ T subsets in the microenvironment of AML. We currently demonstrated that elevated BMP2 induced an immunosuppressive role of γδ T cells through turning a fraction of effector Vδ2 cells into a regulatory subset. Moreover, our unpublished preliminary data showed that Reg-Vδ2 cells also suppressed the cytotoxic ability of conventional CD4^+^ and CD8^+^ αβ T cells against AML cells. The complex regulation and mechanism in this regard await further well-designed experiments. Accordingly, the negative impact of Reg-Vδ2 cells should be considered in the implementation of effector T cells-based immunotherapy for AML. Inhibition of Reg pathway thereby preventing the emergence of Reg-Vδ2 cells may serve as a novel strategy to enhance the efficacy of Vδ2 cells-based immunotherapy. Since the functional complementarity of BMP receptors and ligands during biological processes, blockade of BMP signaling with highly selective compounds might have little toxicity and thus is an exciting prospect for therapeutic means (49, 50). The blocking effect of BMP inhibitor on the emergence of immunosuppressive γδ T subset in our study, along with previous reports showing that BMP inhibitors blocked the BMPs-induced oncogenes expression in AML cells (25, 26), highlights the application significance of BMP inhibitors in the treatment of hematopoietic malignancies. Besides k02288 used in our experiments, other selective inhibitors of BMP receptor kinases have been developed. The safety and efficacy of these inhibitors will need to be carefully evaluated and compared in pre-clinical models of AML.

In summary, combination of immunophenotypical and functional data in the present study identified an immunosuppressive subset of γδ T cells that was correlated with dysregulated BMP2 in AML, leading to the functional impairment of γδ T cells in elimination of AML. Inhibition of BMP pathway significantly blocked the emergence of Reg-Vδ2 cells and enhanced the anti-AML effect of effector Vδ2 cells. These findings shed new lights on the immunosuppressive mechanism in the context of leukemia and suggest potential targets for future studies to improve the immunotherapeutic strategies in the treatment of AML and other hematopoietic malignancies.

## Data availability statement

The raw data supporting the conclusions of this article will be made available by the authors, without undue reservation.

## Ethics statement

The studies involving human participants were reviewed and approved by The Ethics Committee of Peking University People’s Hospital. The patients/participants provided their written informed consent to participate in this study. The animal study was reviewed and approved by The Animal Use Protocol & Ethic Review Committee of Peking University People’s Hospital.

## Author contributions

SL designed and performed the research, analyzed data, and wrote the manuscript; TD, KY, HG, NW, and RL performed the research; YC and LHa collected the blood samples; LHu and TZ provided samples; QJ provided samples and reviewed the manuscript; X-JH interpreted data and revised the manuscript; JL supervised research and wrote the manuscript. All authors contributed to the article and approved the submitted version.

## Funding

This study is supported by the National Natural Science Foundation of China (Grant No. 82270171) and the Foundation for Innovative Research Groups of the National Natural Science Foundation of China (Grant No.81621001).

## Acknowledgments

We thank all patients and investigators involved in the study.

## Conflict of interest

The authors declare that the research was conducted in the absence of any commercial or financial relationships that could be construed as a potential conflict of interest.

## Publisher’s note

All claims expressed in this article are solely those of the authors and do not necessarily represent those of their affiliated organizations, or those of the publisher, the editors and the reviewers. Any product that may be evaluated in this article, or claim that may be made by its manufacturer, is not guaranteed or endorsed by the publisher.
