# Supplementary material for: Identification of the immunosuppressive effect of γδ T cells correlated to bone morphogenetic protein 2 in acute myeloid leukemia
Source: Front Immunol. 2022 Oct 17;13:1009709. doi: 10.3389/fimmu.2022.1009709 (PMC9618638; doi:10.3389/fimmu.2022.1009709)
Supplement: Supplementary file 1 [file DataSheet_1.pdf]

Supplementary Table 1. Clinical characteristics

	AML patients	Healthy donors	*P* value
Subjects, n	62	51	N/A
Age, y	52 (18-65)	43 (18-58)	0.243
Gender (F/M)	37/25	28/23	0.577
Diagnosis, n (%)			
M0	3 (4.8)	N/A	
M2	35 (56.5)	N/A	
M4/5	22 (35.5)	N/A	
M6	2 (3.2)	N/A	

Staging was based on French-American-British (FAB) Classification. N/A, not applicable.

A

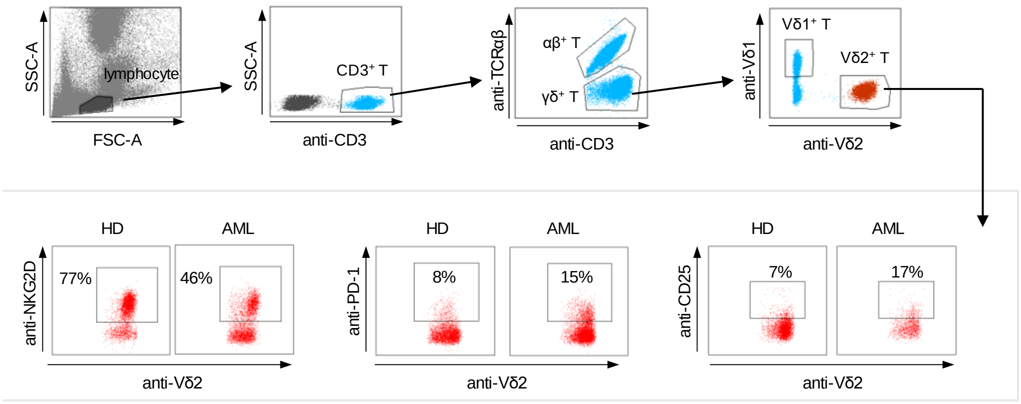

B

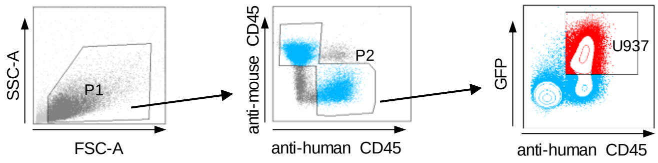

Supplementary Figure 1 Representative images of the gating strategies by flow cytometry analyses

A Gating strategies for γδ T, Vδ1$^+$ and Vδ2$^+$ T cells, and expressions of NKG2D, PD-1, and CD25 on Vδ2$^+$ T cells. **B** Gating strategies for human CD45$^+$ cells, and GFP$^+$ U937 cells in the peripheral blood of mice.

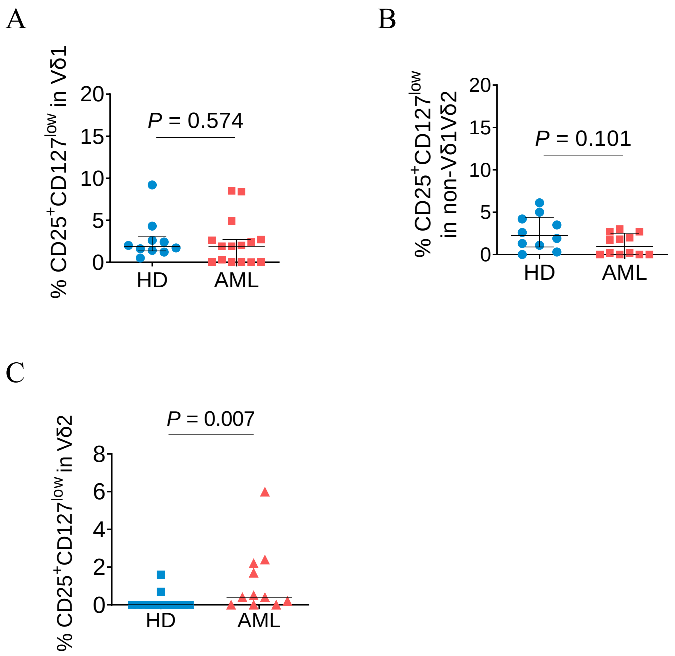

Supplementary Figure 2 The proportions of CD25$^+$CD127^{low} fraction in Vδ1 and non-Vδ1Vδ2 cells of bone marrows and the frequency of CD25$^+$CD127^{low}Vδ2$^+$ T cells in peripheral blood

Bone marrow samples were collected from healthy donors (n = 10) and AML patients at diagnosis (n = 15) and detected by flow cytometry analyses. **A** The percentage of CD25$^+$CD127^{low} fraction in Vδ1$^+$ T cells. **B** The percentage of CD25$^+$CD127^{low} fraction in non-Vδ1Vδ2 cells. **C** The frequency of CD25$^+$CD127^{low} Vδ2 cells in peripheral blood samples from healthy donors (n = 13) and AML patients at diagnosis (n = 11). *P* values are indicated on the graphs.
